# Interactive effects of light and nutrients shape phytoplankton thermal traits

**DOI:** 10.1038/s41598-025-27601-w

**Published:** 2025-11-18

**Authors:** Anna Lena Heinrichs, Miriam Gerhard, Maren Striebel

**Affiliations:** 1 Institute for Chemistry and Biology of the Marine Environment (ICBM), Carl-von-Ossietzky University of Oldenburg, School of Mathematics and Science, Ammerländer Heerstraße 114-118, 26129 Oldenburg, Germany; 2 Departamento de Ecología y Gestión Ambiental, Centro Universitario Regional del Este, Universidad de la República, Tacuarembó s/n, 20000 Maldonado, Uruguay

**Keywords:** Growth, Response surface, Resource limitation, Specialist-generalist, Thermal sensitivity, Trade-off, Ecology, Ecology, Environmental sciences

## Abstract

The variable character of aquatic systems forces organisms to constantly adjust to their changing environment. We investigated how resource availability shapes the temperature sensitivity of growth for the freshwater phytoplankton species *Scenedesmus armatus (Chodat)*. We used a gradient design of light and nutrients, to assess their interdependent effects on numerous thermal traits, determined from thermal performance curves (TPCs). Building on previous studies that demonstrated resource-dependent temperature sensitivity in growth for a single resource type, we advance this understanding by revealing the interdependent effects of multiple resources on thermal traits, using response surfaces. We showed that most resource effects on thermal traits highly depend on the availability of other resource that can even change the direction of the effect, and that thermal traits can differ in their resource dependence. Our study provides important knowledge of how organisms cope in their changing and multifactorial environment, especially in the face of climate warming.

**Supplementary Information:**

The online version contains supplementary material available at 10.1038/s41598-025-27601-w.

## Introduction

**Fig. 1:**
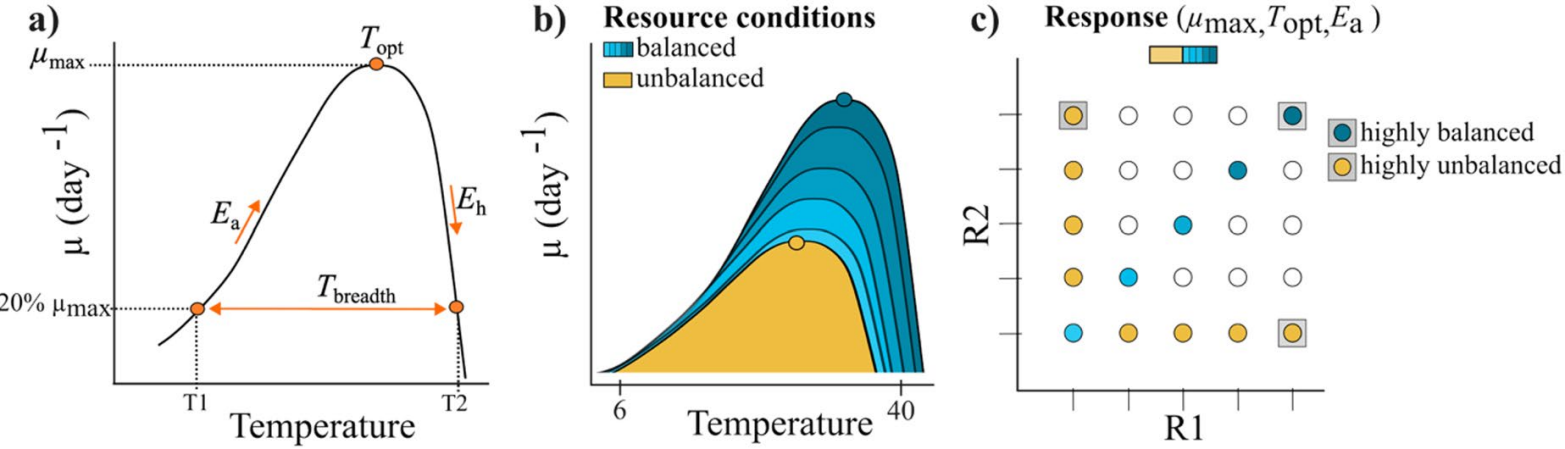
(**a**) Schematic TPC showing the estimated thermal traits in this study (i.e., *T*_opt_, *µ*_max_, *T*_breadth_, *E*_a_ and *E*_h_) (**b**,**c**) theoretical shapes of the TPC in dependence of the availability of multiple resources. The shape of the TPC is expected to depend on the availability of both resources, light and nutrients. Higher values of *T*_opt_, *µ*_max_ and *E*_a_ are expected when resources are supplied together (defined as *balanced resource conditions*, turquoise colors) compared to conditions where only one resource is supplied while the other keeps at low level (defined as *unbalanced resource conditions*, yellow colors). Further, at balanced resource conditions, we expect increasing values of these traits with increasing supply, leading to maximum expected trait values at highest level of both resources together (defined as *highly balanced resource conditions*). In contrast, trait values are expected to stay unchanged at unbalanced resource conditions although one resource is at highest level because responses are restricted by the limited resource (defined as *highly unbalanced resource conditions*).

 Temperature is a fundamental driver that affects the metabolism and growth of organisms^1,2^, shaping consequently resource competition^3^ and community structures and dynamics^4^. Therefore, organismal thermal responses holds significant ecological implications. The response in performance (i.e., growth rate) of an ectothermic organism along a gradient of temperatures – the so called *thermal performance curve* (TPC,^5^) – is highly nonlinear and can be generally described as a unimodal left-skewed function^6^. Thereby growth declines more rapidly above the optimum temperature than below^5,7^, meaning that small rises in temperature above the optimum translate into strong declines in growth. Thermal traits, derived from the TPC, are often used to evaluate the thermal sensitivity of an organism’s performance. For phytoplankton populations, a variety of thermal traits – the temperature optimum (*T*_opt_), maximum growth rate (*µ*_max_), thermal breadth (*T*_breadth_), activation energy (*E*_a_) and deactivation energy (*E*_h_), (Fig. 1a) - have been widely used to evaluate potential changes in species growth and persistence under expected climate change scenarios^6,8–10^.

Despite its widespread use in assessing population vulnerability to climate change, the shape of the thermal performance curves is highly context-dependent. For instance, thermal adaptation and acclimation shape the temperature preferences and tolerances in phytoplankton^6,11–13^ leading to different thermal responses across regions differing in temperature conditions^6^. In addition to the thermal context, other environmental factors, such as the availability of resources, can influence the response to temperature. Both resources, light and nutrients, are key drivers in aquatic ecosystems that influence the primary producer’s growth^14,15^. When evaluated independently, it has been shown that the availability of nutrients^9,10,16^ and light^17,18^ influence phytoplankton thermal responses and associated traits.

While the impact of light intensity on thermal traits has rarely been studied (but see Edwards et al. 2016^18^), the influence of nutrient availability on thermal traits is often reported. For instance, it has been shown that low nutrient concentrations shift the temperature optimum, *T*_opt_, to lower temperatures^9,10^, suggesting that phytoplankton living under low nutrient conditions are less tolerant for higher temperatures^19^. Further, nutrient limitation can suppress the positive response in photosynthetic rate with rising temperature, the *activation energy*. As a consequence, the TPC becomes flatter, and the thermal sensitivity is reduced^20^. Such flattening of the TPC, accompanied by a decline in maximum growth rate, has also been reported in the study by Aranguren-Gassis and Litchman (2020)^21^ that tested for the influence of the nutrient availability on the TPC of six marine phytoplankton species.

The metabolism and resource requirements of phytoplankton are highly temperature-dependent^3,12,13^. A previous study showed interactive effects of temperature, light and nutrients on phytoplankton growth^22^. This work demonstrated that the positive effect of resource supply (e.g., nutrients) on growth responses to rising temperature highly depends on the availability of the other resource (e.g., light) and was inhibited, when that is limited^22^. This highlights the need to consider multiple resources to improve the predictive power of models designed to capture the consequences of temperature changes and the transfer to nature^23^.

To go a step further and determine thermal traits and their potential consequences for the thermal sensitivity of a phytoplankton population, this study investigates the role of multiple resources in shaping the thermal performance curve. We focus on five thermal traits which are the maximum growth rate (*µ*_max_), the temperature optimum (*T*_opt_, the temperature at *µ*_max_), the thermal breadth (the width of the TPC at which the growth is higher than 20% of *µ*_max_), the activation-, and deactivation energy (steepness of the increasing and decreasing part of the TPC).

We hypothesize that light and nutrients interactively shape the response to temperature; in other words, the shape of the TPC (i.e., the thermal traits) depends on the availability of both resources, light and nutrients. We expect positive effects on thermal traits when nutrients and light are supplied together while the limitation of one resource restricts the effects of the other resource.

Further, we also test for relationships between thermal traits as there have been debates about the role of thermodynamic constraints affecting the thermal response and thermal traits of ectotherms^24^: The *hotter-is-better hypothesis* posits a positive relationship between *T*_opt_ and *µ*_max_ due to the influence of thermodynamics on enzyme kinetics whose activity rate increases with rising temperature until the temperature optimum^5,25^. In contrast, the *specialist-generalist trade-off* is thermodynamically weaker, but presents a trade-off between high performance (*µ*_max_) and a high thermal tolerance range (*T*_breadth_) as high performance comes with a cost, reducing the thermal tolerance range.

## Methods

We conducted a laboratory experiment using a non-axenic monoclonal culture of *Scenedesmus armatus (Chodat)* (hereafter *Scenedesmus)*, isolated in July 2020 from an oligotrophic lake (Grafschaftssee, Germany, 53°33005″ N; 7°58049″ E). Before the experiment was started, the species was cultured in a reduced culture medium ( ¼ WC, Guillard and Lorenzen 1972), at 18 °C on a 12:12 light-dark cycle with 70 µmol photons m^- 2^ s^- 1^ light intensity. As the species originated from oligotrophic conditions, we kept the nutrient concentrations in the medium reduced instead of using the full medium to ensure nutrient conditions closer to nature.

### Experimental design and sampling

To test for the combined effect of light and nutrients on thermal traits of the TPC, we used 8 constant temperatures ranging from 7 °C to 43 °C, combined with 5 levels of light intensity and 5 levels of nutrient concentrations (Table 1). We incubated *Scenedesmus* for one week in 24-well plates (SARSTEDT AG & Co.KG) with a sample volume of 2 ml, using 4 replicates for each treatment, resulting in 800 units in total. For the nutrient additions, we used nitrogen (N, as NaNO_3_) and phosphorus (P as K_2_HPO_4_) in different concentrations (Table 1) but at the same molar ratio (N: P of 16:1, matching the Redfield ratio) at the beginning of the experiment as a single addition. To avoid limitation by other elements, we added all other nutrients and vitamins according to ¼ WC growth medium^26^. For the light intensity gradient (33–250 µmol photons m^− 2^ s^− 1^, Table 1), we covered each well plate with light filter foils that reduced the intensity but retained the full light spectrum (LEE Filters no. 209, 210, 211, 298 reduce light by 30%, 49%, 77% and 87%) and located all well-plates below LED panels (Solar Stringer Fresh Sun Strip 35, Econlux GmbH). To establish the different temperature conditions (Table 1), we used different heat mats on which the well plates were placed, all of them exposed in temperature controlled climate chambers (6 °C chamber for temperatures below 18 °C, and 18 °C chamber for temperatures at or higher that 18 °C). To ensure a regular heat distribution along the mat and reduced variability, the mats were covered with sand. To track the temperatures during the experiment, continuous data logger (Hobo Pendant ^®^, Onset, Bourne, MA, USA) were exposed on the mats (Fig. S1). In order to avoid an evaporation of samples that were incubated at high temperatures, we covered all well plates with airtight foils under the lid of the plates which were removed and renewed daily during sampling.

**Table 1:** Light intensities, nutrient concentrations and temperature conditions used in the experiment. Nitrogen and phosphorus were added at the beginning of the experiment to all units in a ratio of 16:1 but with different concentrations. Each light level (5 levels) was combined with each nutrient concentration (5 levels) with each temperature level (8 levels) and with 4 replicates for each treatment resulting in 800 units in total. Resource levels were chosen based on previous work that showed that these concentrations were below saturating conditions for this species^22,27^. See Fig. S2 for an overview of sample conditions before and during the experiment.

Light intensity		Nutrients (µmol L^− 1^)			Temperature
(µmol photons m^− 2^ s^− 1^)		N	P		°C
33		5.0	0.3		7
58	**×**	16.5	1.0	**×**	10
128		28.1	1.8		18
170		39.7	2.5		22
250		51.2	3.2		27
					30
					38
					43

The optical density (OD, absorbance at 440 nm) was measured daily using a microplate reader (Synergy H1, BioTek instruments) to track the biomass development over time. We used optical density (OD) as a proxy for biomass because, unlike pigment-based measurements, it is not influenced by differences in light conditions during growth. To ensure homogenized conditions, we resuspended each sample with a 1 ml pipette prior to the measurements. For daily measurements, the lid and the airtight foils of the well plates were removed, enabling gas exchange, and a new airtight foil was added afterwards.

### Data analysis

We performed the statistical analysis as well as the Figures using R, version 4.1.1 (the R Foundation for Statistical Computing Platform)^28^. The conceptual figure (Fig. 1) was created with the software *Inkscape*.

#### Growth rates

The net growth rates *r* (day^− 1^) were defined as the maximum growth rate given by the OD measurements over time. Linear models were fitted to the exponential part of the growth curve at a logarithmic scale using the R package *‘growth rates’* and the *‘fit_easylinear’* function^29^. For the samples exposed to temperatures above 30°C, the R package could not be applied as biomass was decreasing over time (see Figure S3 for OD trend over time). For those treatments, we calculated the linear slope between the start and the end OD using the following equation:

1 $$\:\text{r}\:\left({\text{d}\text{a}\text{y}}^{-1}\right)=\frac{\text{l}\text{n}\:{\text{O}\text{D}\text{t}}_{\text{e}\text{n}\text{d}}\:-\text{l}\text{n}\:{\text{O}\text{D}\text{t}}_{\text{s}\text{t}\text{a}\text{r}\text{t}}\:}{\left({\text{t}}_{\text{e}\text{n}\text{d}}-\:{\text{t}}_{\text{s}\text{t}\text{a}\text{r}\text{t}}\right)}\:\:\:\:$$

where OD is the optical density at the end (*t*_end_) and at the beginning (*t*_start_) of the experiment. The experiment ran for 13 days but, depending on the treatment conditions, the samples reached latest after 7 days the stationary phase thus the first 7 days were used for growth rate calculations.

#### TPC fitting

For fitting the TPCs, we used Generalized Additive Models (GAM) with temperature as smooth function for each resource combination (in total 25 resource combinations). We interpolated the predicted growth rates from the GAM across the temperature range of 7 °C to 43 °C, in temperature steps of 0.5 °C (hereafter called *interpolated growth rates*) for each resource combination.

#### Thermal traits

In order to test how light and nutrients shape the TPC of *Scenedesmus*, we determined the following thermal traits for each resource combination using the interpolated growth rates: the maximum growth rate (*µ*_max_), the temperature optimum (*T*_opt_), the activation (*E*_a_), and deactivation energy (*E*_h_), and the thermal breadth (*T*_breath_) (Fig. 1a). We defined *µ*_max_ as the peak growth rate within the TPC, *T*_opt_ as the corresponding temperature to *µ*_max_, and the thermal breadth, *T*_breadth_, as the range of temperatures at which growth is greater than 20% of *µ*_max_, because previous work has indicated that species commonly appear or even dominate in the field at temperatures in this thermal range^7,30^. The activation energy, *E*_a_, which characterizes here the strength of the positive response in growth to temperature below *T*_opt_, was determined with the interpolated growth rates below *T*_opt_ using a linear model to estimate *E*_a_ based on the Arrhenius equation (Eq. 2, see Figure S4 for linear regressions). The deactivation energy, *E*_h_, which characterizes how strong growth decreases above *T*_opt_, was determined with the interpolated growth rates above *T*_opt_ using again a linear model to estimate *E*_h_ based on the Arrhenius equation (Eq. 2, see Figure S5 for linear regressions). The activation and deactivation energy can be calculated with the slope of a linear model, with growth as response variable and temperature as independent variable, and the Boltzmann’s constant (Eq. 2).

2 $$\:r=A\:\times\:{e}^{-\frac{E}{RT}}\:$$

Arrhenius function, where *r* is the growth rate, *A* the y-intercept, *E* the activation or deactivation energy (eV), *R* the Boltzmann’s constant (8.62 × 10^− 5^ eV K^− 1^) and *T* the temperature (in K). The function can be solved with a linear model using the following equation (Eq. 2.1):

2.1 $$\:\text{ln}r=-\frac{E}{R}\:\frac{1}{T}+\text{ln}A\:\:$$

where $\:-\frac{E}{R}\:$ is the slope of the linear model, $\:\frac{1}{T}$ is the x-intercept, and $\:\text{ln}A$ is the y-intercept. Hence, the activation energy *E*_a_ and deactivation energy *E*_h_ equal the slope of the linear model multiplied with *R* and a factor of *-1*. For the deactivation energy, we did not use the factor of -1 to avoid negative values and therefore higher values presenting a steeper slope.

To test for the resource effects on the thermal traits, *µ*_max_, *T*_opt_, *E*_a_, *E*_h_ and *T*_breadth_, we used linear models with light and nutrients as main effects and a linear pairwise two-way interaction between light and nutrients. As previous studies have shown saturating patterns in some thermal traits along a resource gradient^9,10^, we compared models that included only the linear terms and models that additionally included quadratic terms (hereafter *polynomial model*). Different models were compared by AIC and the best model was selected (see Appendix Eqs. 1–5 for model functions used for the different thermal traits). The polynomial model improved the AIC and *R* for all thermal traits (see Figure S6 for model validation). We then used the predicted thermal trait values of the polynomial model to create the response surfaces for each thermal trait (see Figure S7 for response surfaces of the observed thermal traits).

## Results

**Fig. 2:**
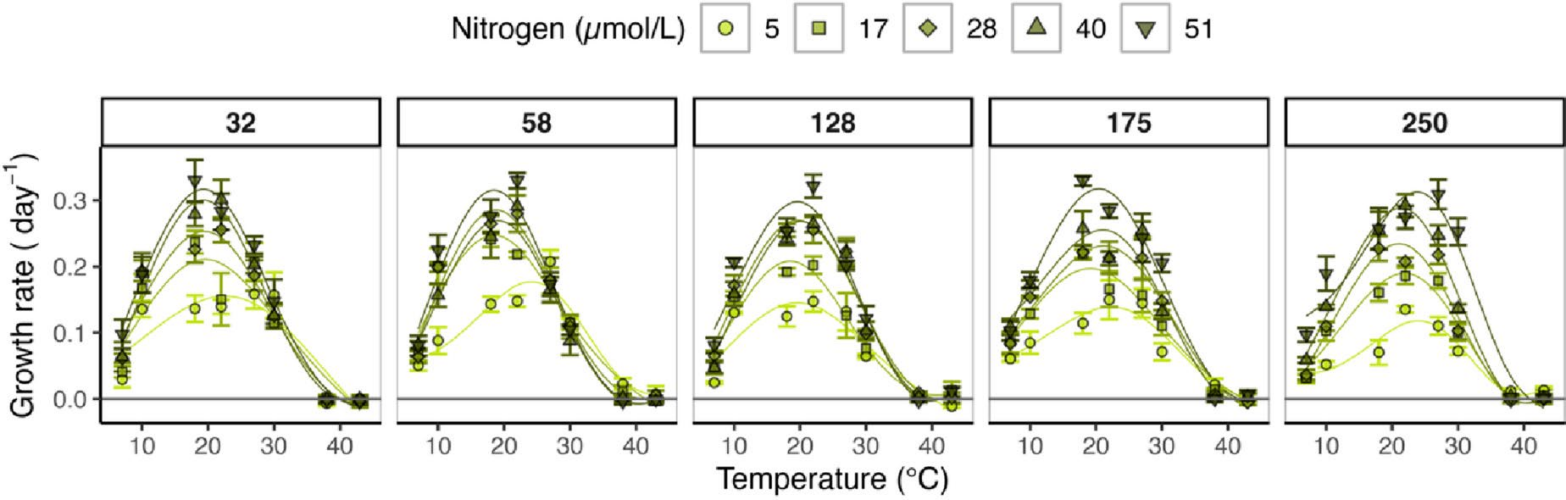
Thermal performance curves at different resource conditions. Different colours and shapes present the different nutrient supply concentrations (only N concentrations are included for simplicity as N and P were always supplied in the same ratio (16:1 ratio, see Table 1 for corresponding phosphorus concentrations), and panel grids the light intensities (in µmol photons m^- 2^s^- 1^). Solid lines present the interpolated TPCs obtained by the GAMs. Data points present the observed mean growth rates of the replicates (*n* = 4) with its standard error as error bar. Horizontal black line presents y-intercept where growth is 0.

**Fig. 3:**
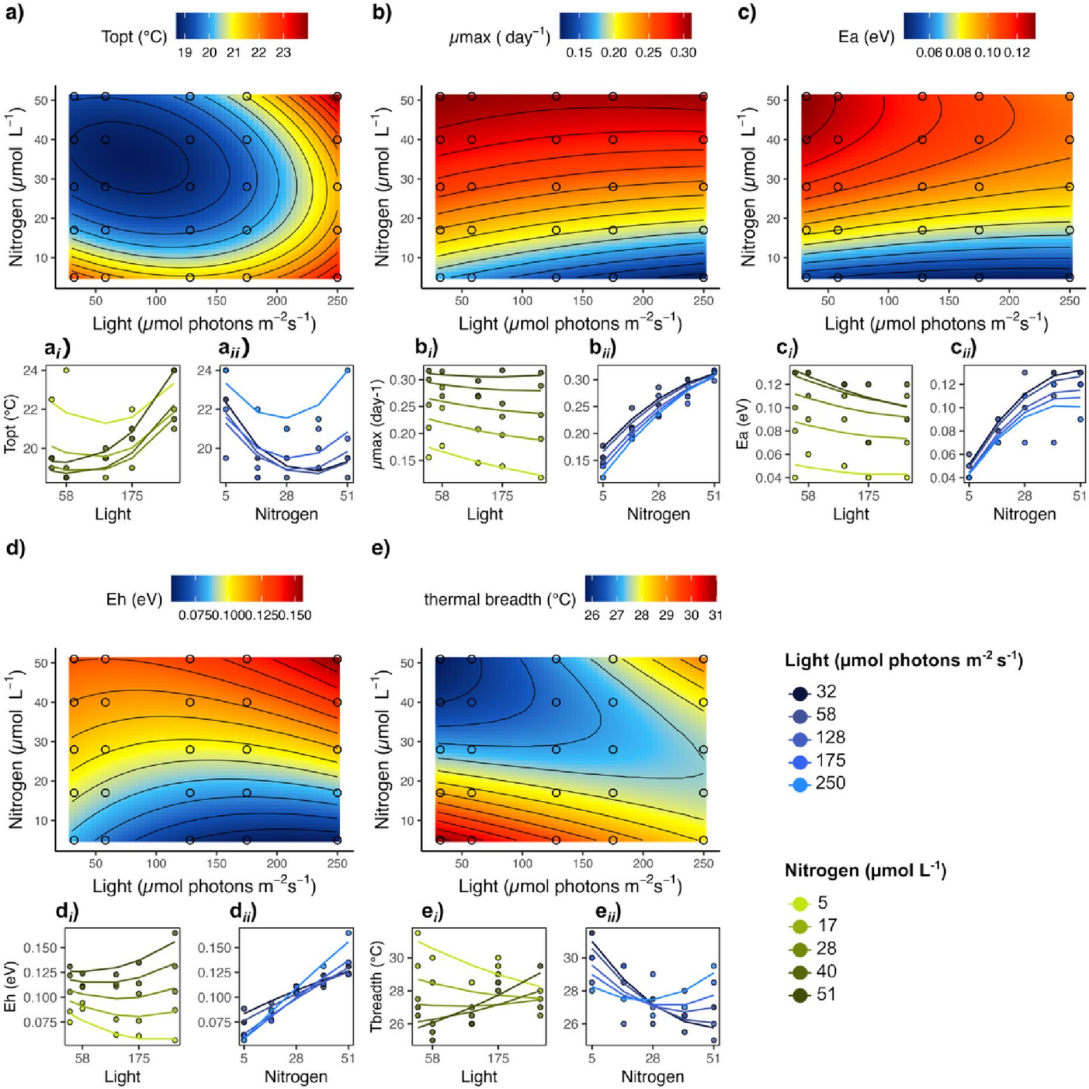
Response surfaces of thermal traits along the resource gradients based on the thermal traits predicted by the polynomial models. Nitrogen and phosphorus were added in a ratio of 16:1 and here only N concentrations are shown for clarity (see Table 1). Circles present the resource treatments used in this study and ggplot ‘geom_contour’ was used for contours and isoclines. Lower plots (**a**_***i,ii***_)–(**e**_***i,ii***_) present observed trait values along either the light gradient for each nutrient level (a_***i***_)–(**e**_***i***_, green colours), or along the nutrient gradient for each light level (**a**_***ii***_)–(**e**_*ii*_, blue colours). Solid lines present the regressions of the polynomials model. Note here; higher values of the deactivation energy, *E*_h,_ present a steeper slope and thus a stronger deactivation energy (see methods).

Within the used temperature range (7–43 °C), the growth rates followed the expected shape of a TPC in all light-nutrient combinations, allowing the estimation of thermal traits under the influence of nutrient supply and light intensity (Figs. 2 and 3). For response variables showing a significant interactive light-nutrient effect (*p* < 0.05), we only focus on this interaction as it dominates then the main effects.

### Temperature optimum (T_opt_)

*T*_opt_ ranged, depending on the resource conditions, from 19 °C to 24 °C. In general, light and nutrients interactively affected the *T*_opt_ in a positive direction (Table 2); meaning the higher the availability of one resource, the more positive the response to the other resource gradient (Fig. 3a_*i*_, _*ii*_ and Table 2). In contrast, lowest *T*_opt_ values occurred at highly unbalanced resource conditions when light intensity was at lowest but nutrient supply at highest level (Fig. 3a). However, these trends were not strictly linear (Fig. 3a), resulting in a more U-shaped response along the nutrient gradient (Fig. 3a_*ii*_, Table 2).

### Maximum growth rate (*µ*_max_)

The maximum growth rate, *µ*_max_, ranged from 0.12 day^- 1^ to 0.32 day^- 1^ depending on the nutrient supply. At all light levels, nutrients had a strong positive effect on *µ*_max_ with a saturating manner at highest nutrient supply concentration (Fig. 3b; Table 2). In contrast, light intensity had a weak and non-significant negative effect on *µ*_max_, which was reduced as nutrient supply increased (Fig. 3b_i_).

### Activation energy (*E*_a_)

The activation energy, *E*_a_, that presents the steepness of the increasing part of the TPC, ranged from 0.04 eV to 0.13 eV. Only nutrients significantly influenced *E*_a_ which resulted in increased *E*_a_ values with increasing nutrient supply, but tended to a saturation shape at highest nutrient concentration (Table 2; Fig. 3c). Light had a weak and non-significant negative effect, which was more pronounced at higher nutrient supply level (Fig. 3c_*i*_).

### Deactivation energy (*E*_h_)

The deactivation energy, *E*_h_, that presents the steepness of the decreasing part of the TPC at temperatures above the *T*_opt_, ranged from 0.06 eV to 0.17 eV depending on the resource conditions. Light and nutrients interactively affected *E*_h_ in a positive direction (Table 2), thus a higher supply of the one resource strengthened the positive effect of the other resource (Fig. 3d): A positive nutrient effect was detected at all light levels, becoming stronger as light intensity increased (Fig. 3d_*ii*_). The direction of the light effect changed from negative to positive with higher nutrient supply (Fig. 3d_*i*_). Consequently, the positive interactive resource effect of light and nutrients resulted in highest *E*_h_ at highest light intensity and highest nutrient supply level (highly balanced resource conditions, see Fig. 1), and lowest *E*_h_ at highly unbalanced resource conditions at lowest nutrient supply and highest light intensity (Fig. 3d).

**Table 2:** Coefficients of the polynomial model testing for the resource effect on the thermal traits. α-terms present the linear coefficients reflecting the slope of the relationship. β^2^–terms present the quadratic coefficients that describe the curvature of the relationship. Bold written values present slopes that differ significantly from 0, and Df the degrees of freedom.

	T_opt_	µ_max_	E_a_	E_h_	T_breadth_
α-Nutrients	**0.0006**	**0.0051**	**0.004**	**0.001**	**-0.32**
α-Light	**0.0007**	-0.0004	-0.0001	**-0.0004**	-0.02
α-Light x Nutrients	**0.00001**	0.000005	-0.000002	**0.000006**	**0.0006**
β^2^-Nutrients	**-0.000008**	**-0.00004**	**-0.00004**	-0.000004	**0.002**
β^2^-Light	**-0.0000008**	0.0000004	0.0000003	**0.000001**	0.00002
df	24	24	24	24	24

### Thermal breadth (*T*_breadth_)

The thermal breadth, *T*_breadth_, ranged from 25 °C to 32 °C depending on the resource conditions (Fig. 3e). In general, light and nutrients interactively affected *T*_breadth_ (Table 2). The light effect changed from a negative to a positive trend as the nutrient supply increased (Fig. 3e_*i*_), and the negative nutrient effect on *T*_breadth_ became weaker as light increased (Fig. 3e_*ii*_). Thereby, the widest thermal breadth was found at lowest supply of both resources, and narrowest thermal breadth occurred at highly unbalanced resource supply with low light and high nutrient conditions (Fig. 3e).

### Correlations between thermal traits

**Fig. 4:**
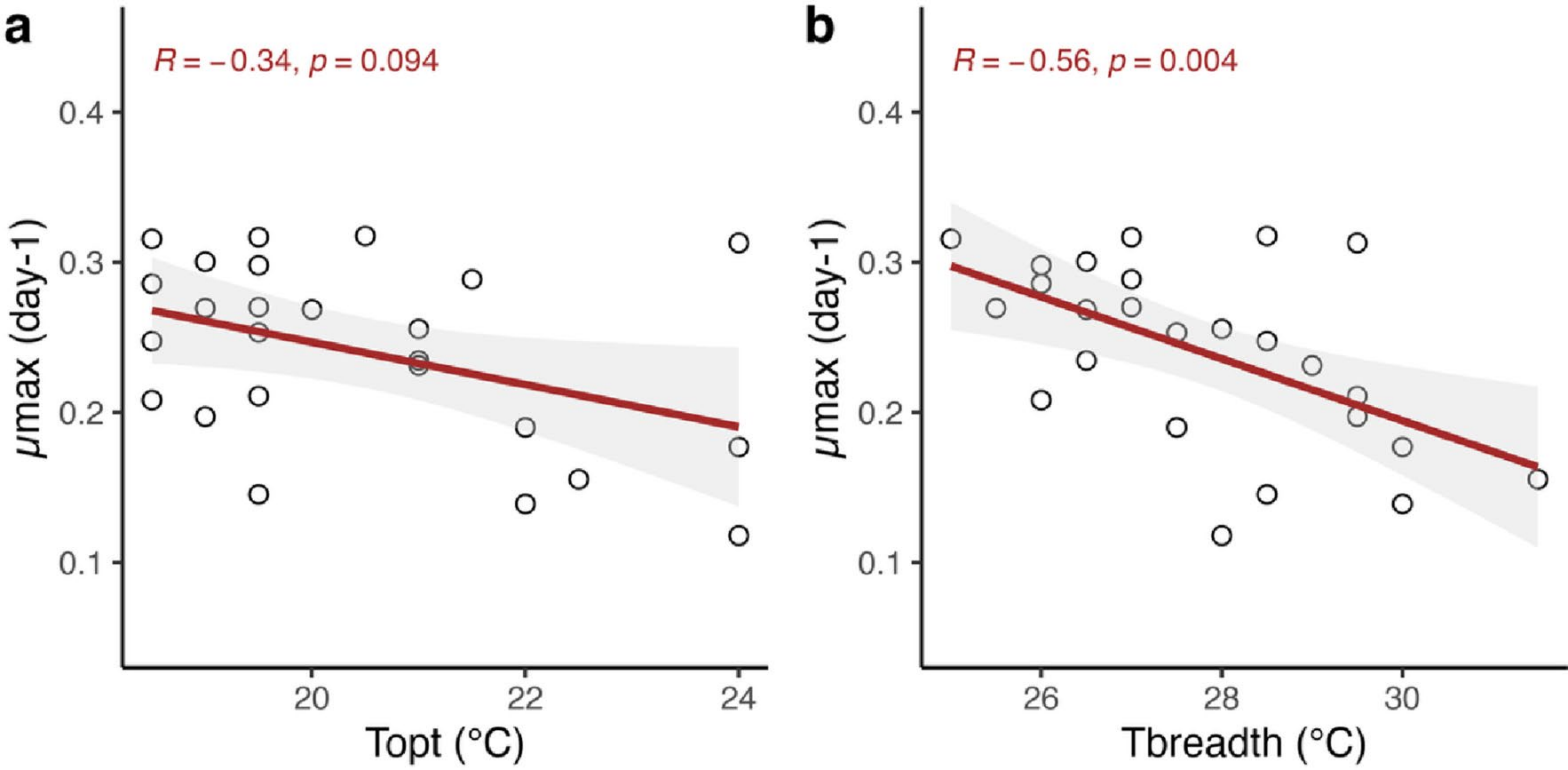
Correlations between thermal traits (*n* = 25) including all resource treatments. *R* gives *correlation of coefficient* (ranges between − 1 and + 1) with p-values, and solid lines present regression lines with 95% confident interval as grey shaded area. a) For the correlation between *µ*_max_ and *T*_opt_, the *Spearman coefficient* was used as *T*_opt_ was not normally distributed. b) For the correlation between *µ*_max_ and *T*_breadth_, the *Pearson coefficient* was used.

To test for relationships between thermal traits that fit into the “*hotter-is-better*” or “*specialist-generalist*” hypothesis, the relationships of interest were those of *µ*_max_ and *T*_opt_ (Fig. 4a) and *µ*_max_ and *T*_breadth_ (Fig. 4b). A positive relationship between *µ*_max_ and *T*_opt_ would support the “the hotter-is-better” hypothesis, and a negative relationship between *µ*_max_ and *T*_breadth_ would fit into the “specialist-generalist” pattern. Both correlations between *µ*_max_ and *T*_opt_, and between *µ*_max_ and *T*_breadth_ showed negative trends but only the negative relationship between *µ*_max_ and *T*_breadth_ was significant (Fig. 4).

## Discussion

In our study, we demonstrate the interdependent effects of multiple resources on phytoplankton’s thermal traits, or in other words, that light and nutrients modulate the temperature sensitivity of phytoplankton. The unimodal shape of the TPC is, among others, a result of the cellular processes photosynthesis and respiration which differ in their sensitivity to temperature^12,13^, and whose balance determines the energy that is available for growth. The fact that photosynthesis strongly depends on light and nutrient availability - and because other studies showed a co-limitation of light and nutrients on phytoplankton growth^31^, which is modulated by temperature^22^ - suggests that the thermal sensitivity of growth is also influenced by these resources. In our study, we were able to identify resource conditions that are optimal or stressful, making phytoplankton more tolerant or more sensitive to temperature changes. Here, we showed that light and nutrients interactively influence the thermal traits *T*_opt_, *E*_h_ and *T*_breadth_, supporting partly our hypothesis. For these thermal traits, we found the highest values when both resources were simultaneously supplied at maximum level. Conversely, lowest thermal trait values did not necessarily appear at lowest resource level of both light and nutrients (low concentrations but balanced supply), but at unbalanced light-nutrient conditions, highlighting the importance of considering both resources together. These findings support our hypothesis that the availability (i.e., nutrient concentration and light intensity) as well as the ratio of the two resources influence the thermal response. Besides these interactive effects on thermal traits, *µ*_max_ and *E*_a_ were mainly affected by the nutrient availability and both increased with higher nutrient concentrations in a saturating manner, independently of light availability, which aligns with other studies^20,21^. As both traits, *µ*_max_ and *E*_a,_ are commonly used to estimate thermal sensitivity, our study suggests that nutrients are the dominant resource type shaping thermal sensitivity and that nutrient rich waters are optimal conditions to maximise growth at the temperature optimum.

Regarding nutrient effects on thermal responses, previous studies have found strong evidence for a positive nutrient effect on *T*_opt_ in marine^10^ as well as freshwater species^9^. We observed such a positive nutrient effect on *T*_opt_ only at highest light level, while lower light intensities diminished this positive nutrient effect, especially at extreme light limitation. Another study, however, did not detect such an increase in *T*_opt_ with increasing nutrient concentration in marine diatoms^21^. Our study can support an explanation for these differences between studies, as we show that the light conditions shape the nutrient effect on *T*_opt_. Therefore, different light conditions and species-specific light preferences might be one explanation as well as the chosen nutrient window that was used to test for the nutrient effect. Although studies that test for the light effect on *T*_opt_ are rare, there is one study showing that increasing light intensity increases *T*_opt_, making phytoplankton exposed to high light conditions more tolerant to higher temperatures^18^. We extend this knowledge with our study by demonstrating that this positive light effect is diminished under nutrient limitation. These findings offer important insights into how phytoplankton respond to stratified conditions, where high light and temperature coincide with nutrient limitation, as well as conditions where light is scarce but nutrients are plenty, such as in eutrophic waters. Our data show that being exposed to such (unbalanced resource) environments imply stressful conditions under warming scenarios as *T*_opt_ is reduced, while having plenty of light and nutrients seem to enhance the tolerance for temperature increase.

The thermal breadth has been proposed to increase in a saturating manner with nutrient concentration^10^. While we could not find such a saturating function of nutrients on thermal breath in our study, we found that the nutrient effect highly depended on the light intensity. Even more, at light limitation (two lowest light level), nutrients had a negative effect on the thermal breadth, while higher light levels diminished or even changed the direction of this effect. Possible reasons for the differences between Thomas et al. (2017)^10^ and our study, in addition to differences in the experimental conditions, could be that the determination of the thermal breadth differed. Since we did not cover the full temperature range down to *T*_min_ where growth equals zero, we used the temperature window at which growth was at least 20% of *µ*_max_. Thus, this approach is limited in its capacity to account for resource effects at thermal extremes (*T*_min_ in this case), and therefore, thermal breadth trends may differ from those reported in previous studies^10^. We found the widest thermal breadth at balanced resource supply, especially when both resources were at low level, accompanied with a flattening of the curve (negative correlation between *T*_breadth_ and *µ*_max_, see Figure S8). Narrowest thermal breadth in contrast was found at highly unbalanced resource supply, especially at low light but high nutrient supply (U-shaped response along the nutrient gradient), suggesting that waters with unbalanced resource conditions are most stressful for phytoplankton living at its thermal limits, like in eutrophic systems with high nutrient but low light availability.

The deactivation energy, *E*_h_, was influenced by the positive interactive effect of both resources (light and nutrients). The highest *E*_h_, thus the steepest slope in growth above *T*_opt_, were found when both resources were at highly balanced conditions, while the lowest *E*_h_ was found under unbalanced resource conditions at highest light but lowest nutrient supply. Although both resources interactively shaped *E*_h_, the nutrient supply always had a positive impact on *E*_h_, independent of the light intensity (only the slope changed with light intensity but not its direction), while the direction of the light effect changed depending on the nutrient supply. All in all, these results suggest that temperatures above the temperature optimum have stronger detrimental effects on growth, at high than at low nutrient supply. This pattern is consistent with the fact that both *µ*_max_ and *T*_opt_ increase with nutrient supply, which leads to a steeper slope of the TPC, assuming that *T*_max_ remains unchanged. This aligns with a study from Aranguren-Gassis and Litchman ( 2020)^21^ who also found flatter slopes at nutrient-limited compared to replete conditions, thus a lower temperature sensitivity under resource limitation. However, these negative impacts on performance (increased *E*_h_) under replete nutrient conditions do not imply a generally worse absolute performance at supra-optimal temperatures compared to lower nutrient condition. As a positive correlation between *µ*_max_ and *E*_h_ exists (Figure S8 and Aranguren-Gassis and Litchman (2020)^21^ and *µ*_max_ strongly increases with nutrient supply, the absolute performance at supra-optimal temperatures can still be higher under replete nutrients compared to lower nutrient conditions.

The negative relationship between *µ*_max_ and *T*_breadth_ suggests trade-offs in thermal acclimation processes and is in accordance with the *specialist-generalist* hypothesis, stating that the ability to perform in a broad range of conditions can be achieved only at the sacrifice of maximal performance^32,33.^ In contrast, the non-significant negative correlation observed between *µ*_max_ and *T*_opt_, does not align with the *hotter-is-better* hypothesis where increasing performance is expected to be linked with higher temperature optima^24,32^. Although the *hotter-is-better* hypothesis is often supported by empirical studies, the thermodynamic effect on maximum performance is still debated as some studies, for example in trees and cyanobacteria, found negative relationships between adaption temperature and maximum performance^34–36^. These studies and our non-significant negative trend suggest that resources might influence thermodynamic constraints, inhibiting the ability to perfectly adjust to the temperature conditions (due to reduced *µ*_max_).

Overall, our study provides important insights into how phytoplankton cope with climate change across environments that differ in multiple resource availabilities *and* temperature conditions. We show that the impact of resources on thermal traits change (among positive, negative, and neutral) in dependence of the other resource, while for some thermal traits (*µ*_max_ and *E*_a_), we could identify dominant nutrient effects. For *T*_opt_, the deactivation energy and the thermal breadth, we found a positive interactive effect of the resources light and nutrients, resulting in highest thermal trait values when both resources were supplied balanced. These complex and interdependent effects on temperature responses highlight the relevance of more complexity in experimental designs to gain a more realistic picture and thus predictive power of how organisms respond to their multifactorial environment.

## Supplementary Information

Below is the link to the electronic supplementary material.

Supplementary Material 1

## Data Availability

The data and R codes used to generate the results in this paper will be archived in Figshare via the following Link https://figshare.com/s/131b95c851a09e661e40.
