# Supplementary material for: Interactive effects of light and nutrients shape phytoplankton thermal traits
Source: Sci Rep. 2025 Nov 18;15:40345. doi: 10.1038/s41598-025-27601-w (PMC12627519; doi:10.1038/s41598-025-27601-w)
Supplement: Supplementary file 1 — Supplementary Material 1 [file 41598_2025_27601_MOESM1_ESM.pdf]

Appendix: Anna Lena Heinrichs, Miriam Gerhard, Maren Striebel. Resource-dependence of phytoplankton thermal traits: interactive effects of light and nutrients.

Linear models used for data analyses (Figure 3 and Table 2)

* present quadratic terms that were additionally added to the linear models to improve the model fit (see Figure S5 for model comparisons).

$T_{opt} \sim Light + Nutrients + \ Light^{*} + Nutrients^{*} + Light \times Nutrients$ Eq 1)

$\mu_{max} \sim Light + Nutrients + Nutrients^{*} + Light \times Nutrients$ Eq 2)

log ($T_{breadth}$) $\sim Light + Nutrients + \ Nutrients^{*} + Light \times Nutrients$ Eq 3)

$E_h \sim Light + Nutrients + Light^{*} + Nutrients^{*} + Light \times Nutrients$ Eq 4)

$E_a \sim Light + Nutrients + Nutrients^{*} + Light \times Nutrients$ Eq 5)

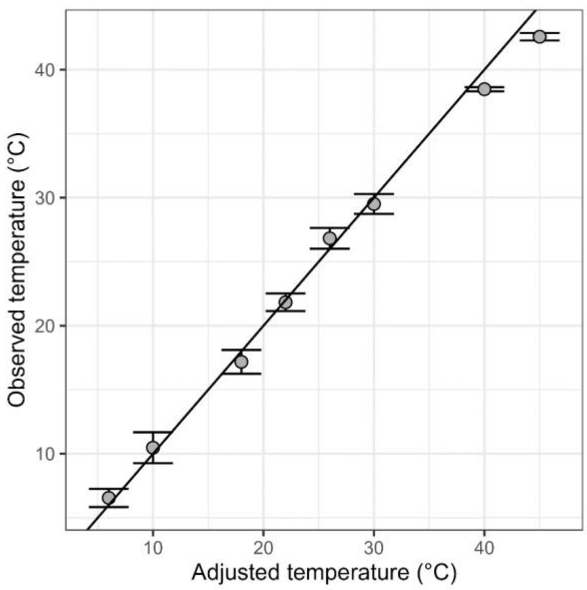

Figure S 1 Observed mean temperatures for each temperature level during the experiment. Measured every 20 minutes with continuous data logger (Hobo Pendant ®, Onset, Bourne, MA, USA). Observed temperatures differed slightly from the adjusted temperatures for the experiment.

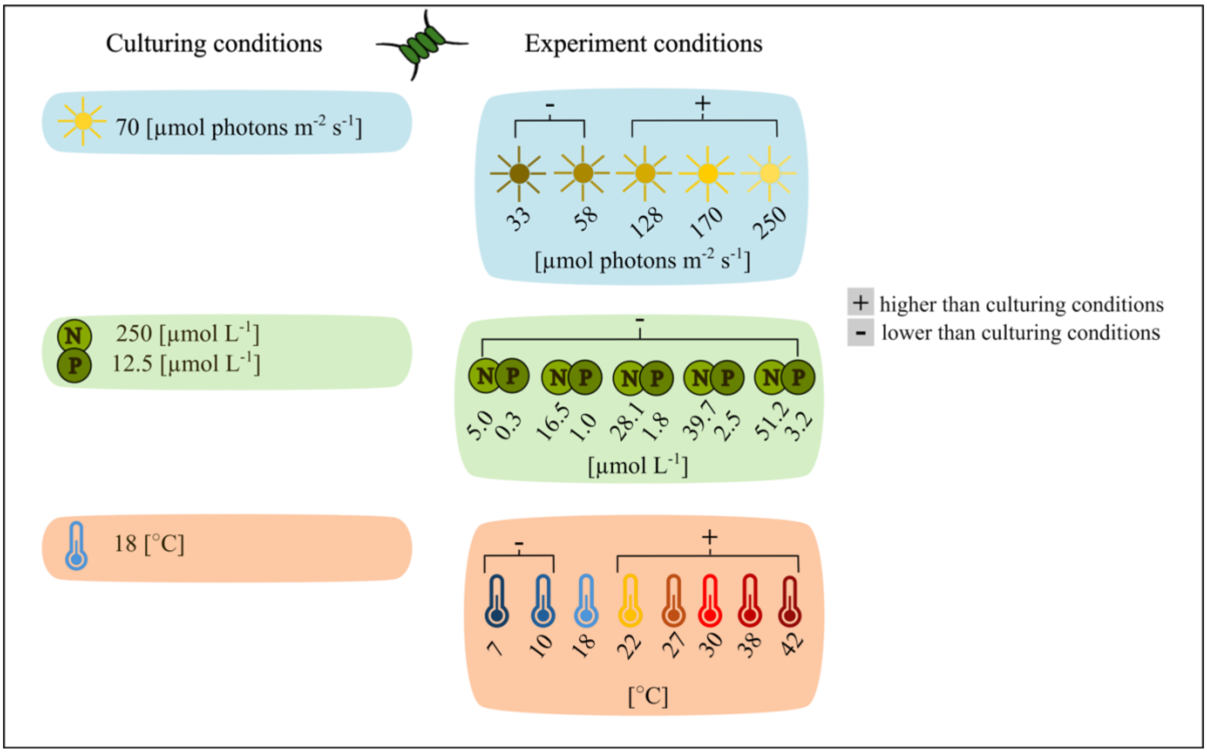

Figure S 2 Resource and temperature conditions before (culturing conditions) and during (experiment conditions) the experiment. Symbols in the experiment conditions (- and +) present treatment levels lower (-) or higher (+) than the culturing conditions. Note: As cultures were kept at high densities before the experiment, the high concentrations of N and P added were depleted quickly, thus actual concentrations are expected to be closer to experimental concentrations.

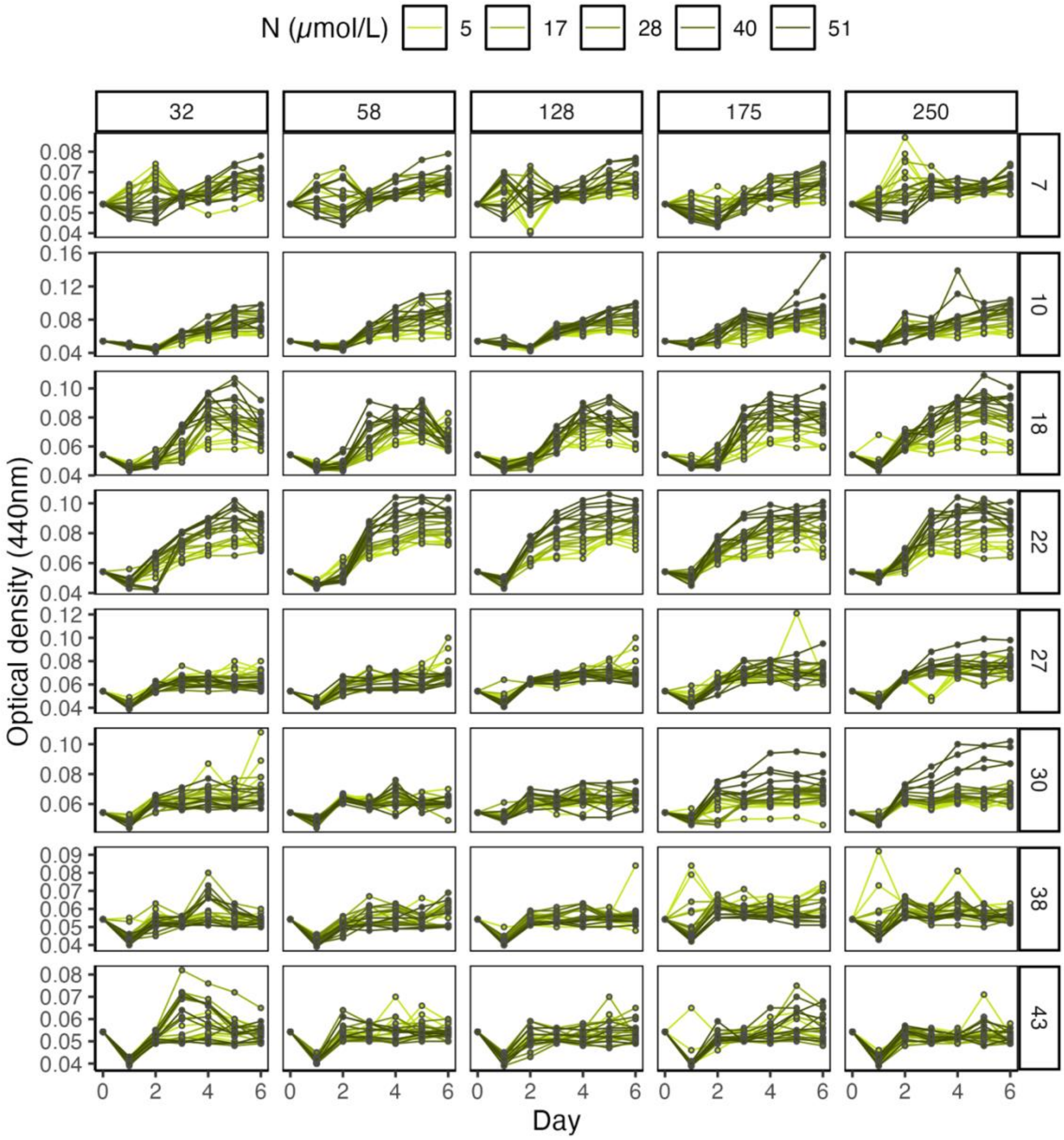

Figure S 3 Incubation growth curves (OD vs time) for each light level (horizontal panels), temperature (vertical panels), nutrient level (colours, *n*=4 of each colour due to replicates).

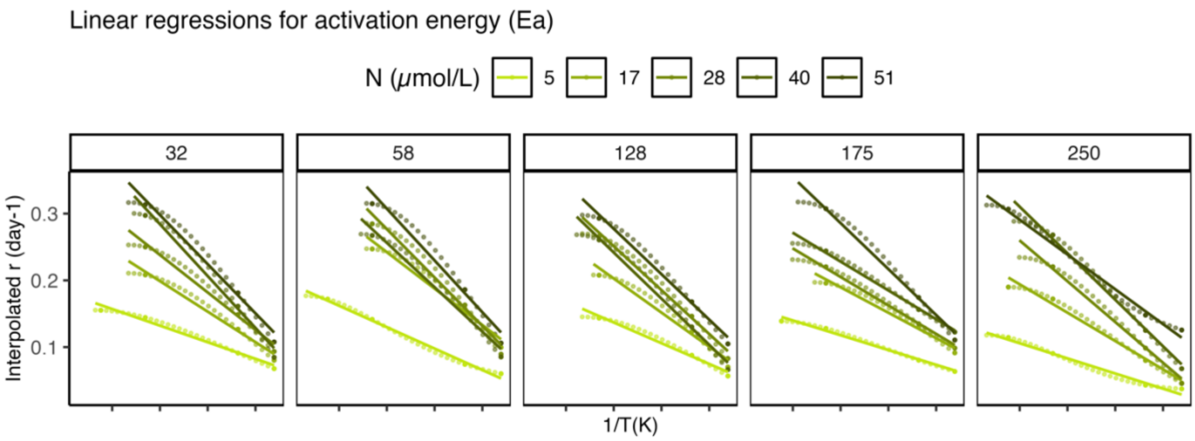

Figure S 4 Linear regressions that were used for the determination of the activation energy, E_a. Interpolated growth rates (coloured circles) below T_{opt} were used to fit linear regressions (coloured lines) of the increasing part of the TPCs. Panel grids present the light intensities and colours the nutrient concentrations.

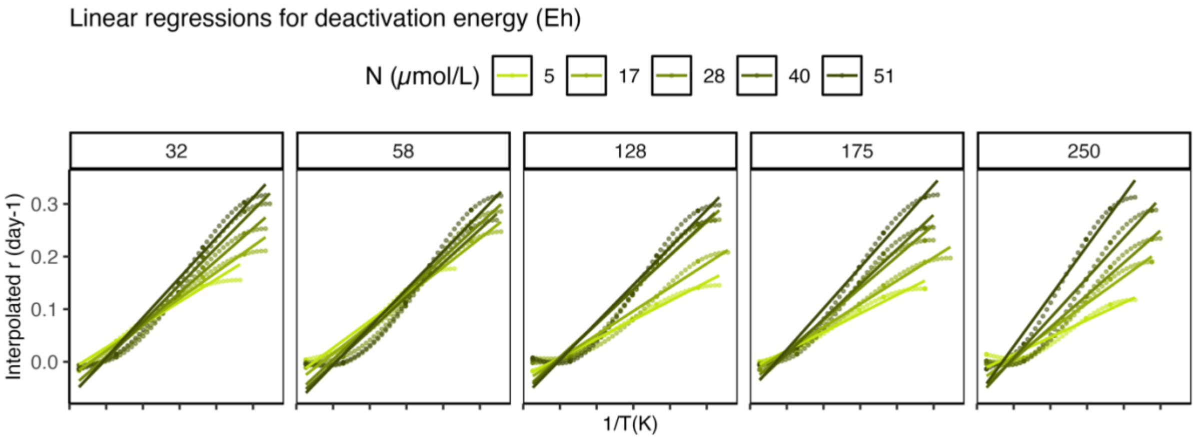

Figure S 5 Linear regressions that were used for the determination of the deactivation energy, E_a. Interpolated growth rates (orange circles) above T_{opt} were used to fit linear regressions (black lines) of the increasing part of the TPCs. Panel grids present the nutrient concentrations in nitrogen (x-panels) and light intensities (y-panels).

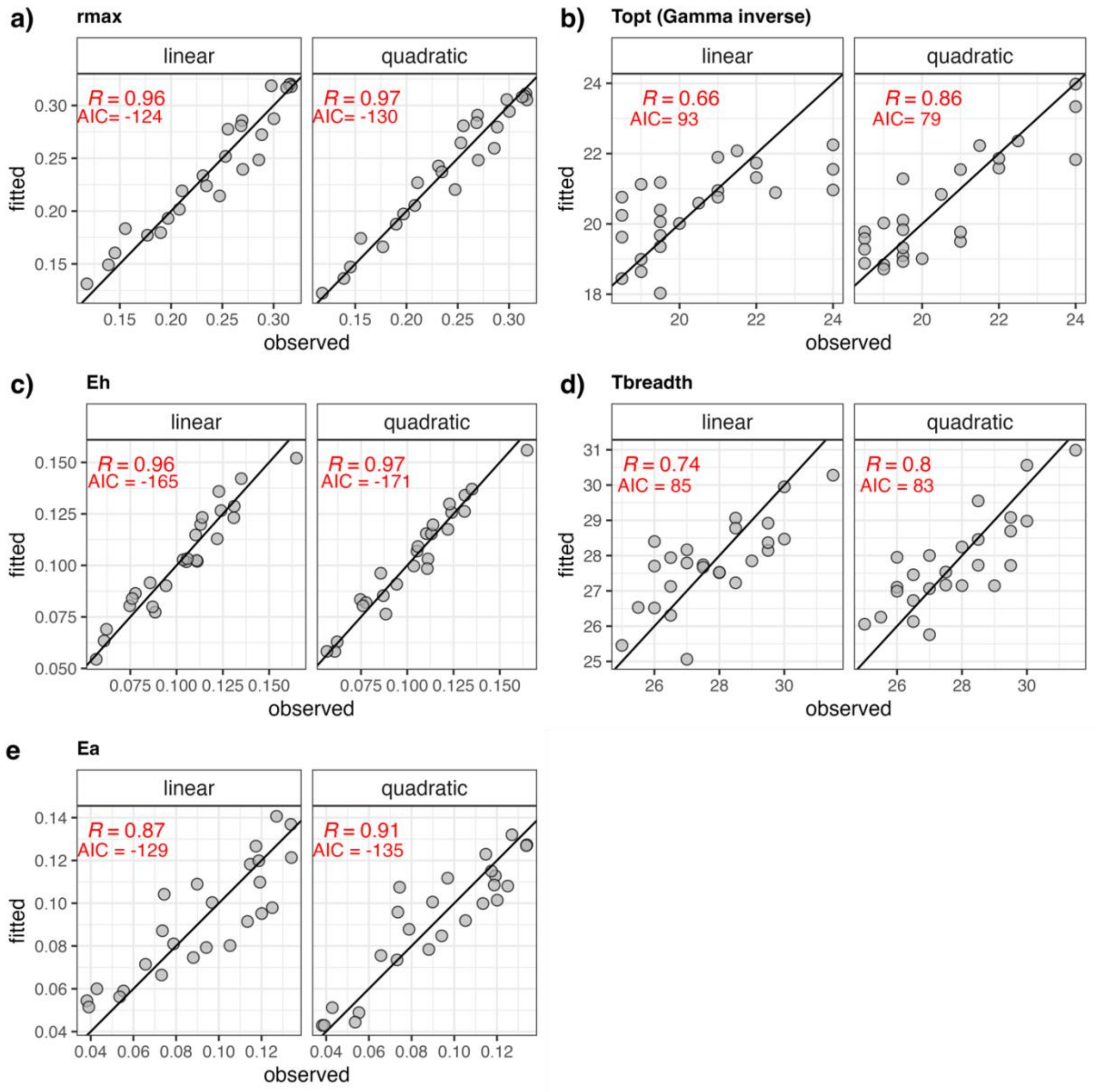

Figure S 6 Model validation for the thermal traits. Comparison of the observed vs predicted trait values of the linear models (*linear* panel) and linear models with quadratic functions (*quadratic* panel). Solid line presents a 1:1 line and red letters the Pearson Coefficient of Correlation *R* and AIC. Based on AIC and *R*, models including quadratic terms lead to better model predictions for all thermal traits.

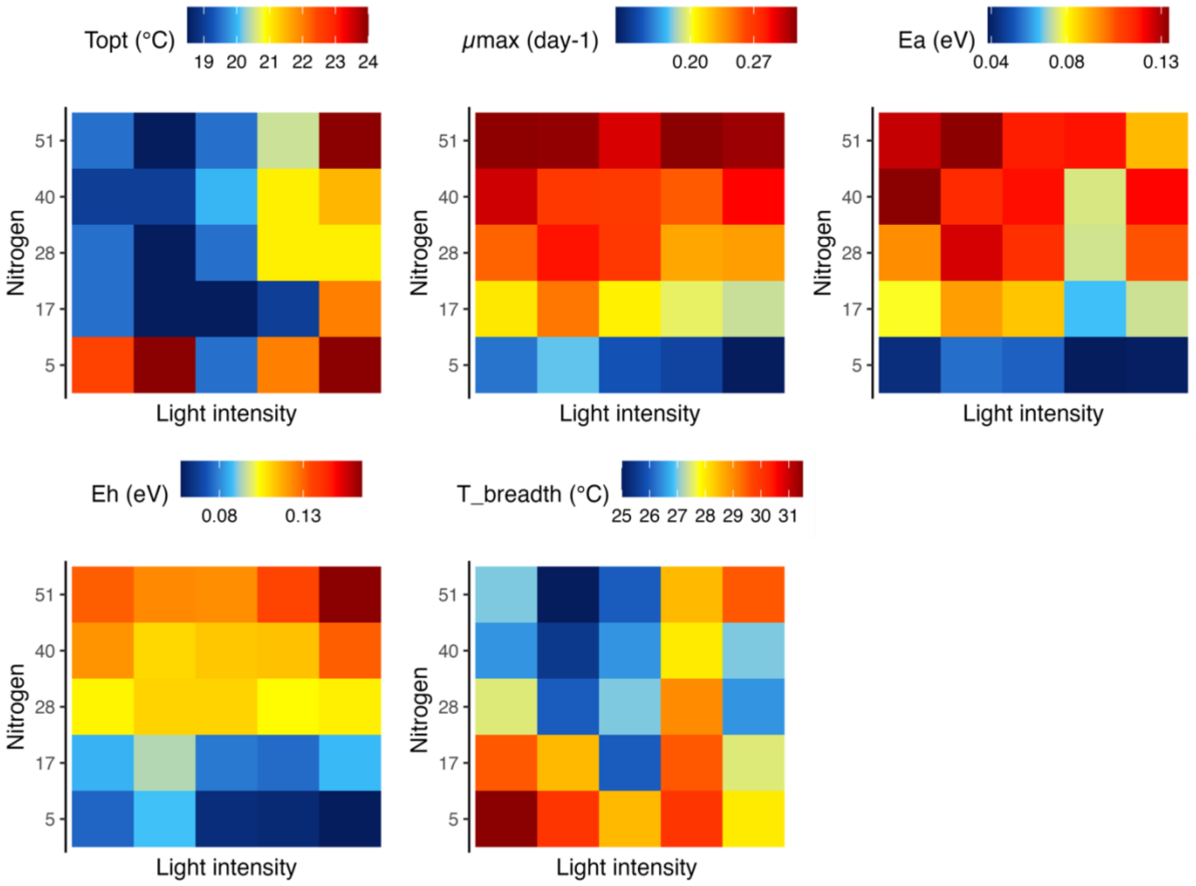

Figure S 7 Observed thermal traits (mean of the replicates, *n*=4) along the five levels of increasing nutrients (here in nitrogen in μmol L^{-1}) and light intensities (μmol photons m^{-2} s^{-1}).

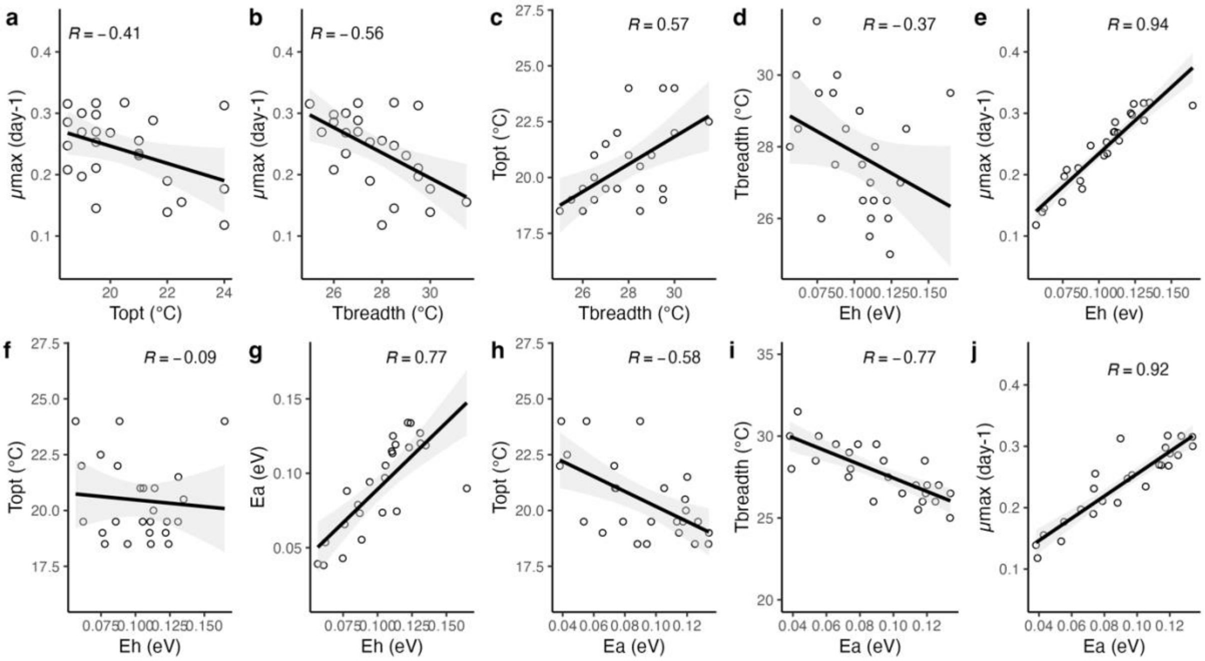

Figure S 8 Correlations between thermal traits (*n*=25) with *R* as Coefficient of Correlation. Panel a-b) present correlations that were included in the manuscript to test for the "hotter-is-better" and "specialist-generalist" hypothesis. Solid line presents linear regression with shaded area as 95% confidence interval.
